# Insights into the Mushroom Tyrosinase Inhibitory, Antibrowning, Antioxidant, and Other Biologically Important Actions of *Juniperus excelsa* subsp. *polycarpos* (Persian Juniper) Essential Oil: In Vitro and In Silico Studies

**DOI:** 10.1021/acsomega.5c06131

**Published:** 2025-11-25

**Authors:** Atiyeh Mahdavi, Mehdi Zanghaneh, Parviz Moradi

**Affiliations:** † Department of Biological Sciences, Institute for Advanced Studies in Basic Sciences (IASBS), 444 Prof. Sobouti Blvd., Gava Zang, Zanjan 45137-66731, Iran; ‡ Julius Kühn Institute (JKI) − Federal Research Centre for Cultivated Plants, Institute for Breeding Research on Horticultural Crops, Erwin-Baur-Str. 27, Quedlinburg D-06484, Germany; § Zanjan Agricultural and Natural Resources Research & Education Centre, AREEO, Zanjan 45195, Iran

## Abstract

Tyrosinase plays a crucial role in the melanin biosynthesis. Abnormalities in pigment synthesis and accumulation are associated with numerous skin diseases. Moreover, since tyrosinase is the main factor for browning of fruits and vegetables, its uncontrolled activity also resulted in serious problems in the food and agriculture industries. Given the importance of tyrosinase inhibition in various fields, identifying safe natural inhibitors is of growing interest in different industries, including agriculture, food, pesticides, pharmaceuticals, and cosmetics. This study investigated tyrosinase inhibitory, antibrowning, antibacterial, and some other biologically important properties of the essential oil isolated from the leaf materials of *Juniperus excelsa* subsp. *polycarpos*, known as Persian juniper, was used for the first time. The maximum tyrosinase inhibitory effect was ∼55%, and kinetic measurements showed a mixed type of inhibition. While fluorescence quenching data revealed decreased intrinsic fluorescence intensity of the enzyme in the presence of increasing amounts of essential oil, molecular docking studies also predicted that the most abundant active compounds bind to the critical binding sites of the enzyme. The *J. excelsa* essential oil also exhibited an efficient antibrowning effect on the tested vegetables and fruits, and it seems that it can decrease tyrosinase-induced hyperpigmentation-related problems in the food and agriculture industries. It exhibited good antioxidant and antibacterial activities and did not show hemolytic activity against human red blood cells in the tested range. These findings suggested that *J. excelsa* essential oil can be considered as a suitable candidate for further investigations on tyrosinase inhibition and antibrowning studies.

## Graphical abstract

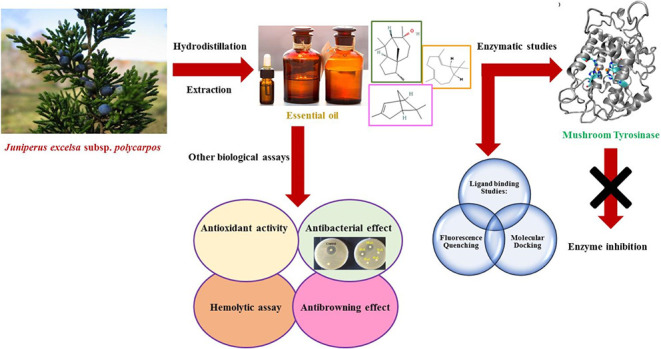

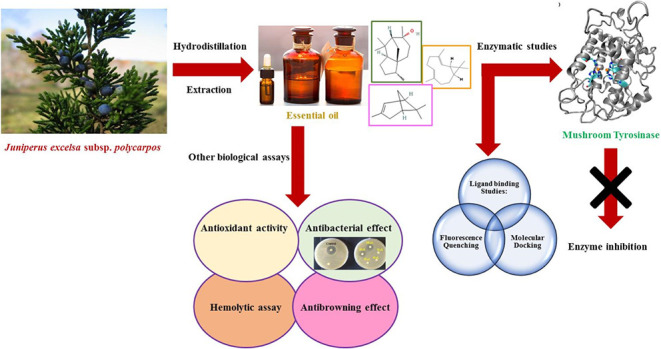

## 1 Introduction

Tyrosinase (EC 1.14.18.1) is the principal enzyme involved in the synthesis of melanin. This enzyme, which contains copper, is present in a diverse array of organisms, from unicellular organisms to mammals. Its functions include both monophenolase (cresolase) and diphenolase (catecholase) activities. Tyrosinase initiates the oxidation of l-tyrosine to L-3,4-dihydroxyphenylalanine (L-DOPA) and subsequently catalyzes the conversion of L-DOPA to quinones. The highly reactive quinones can then undergo a series of nonenzymatic processes, leading to the synthesis of melanin. In addition, tyrosinase is responsible for the generation of flavor compounds that enhance the quality of food processing.^1,2^ Moreover, it is also responsible for enzymatic browning in vegetables and fruits, resulting in undesired color changes and reduction of their taste, quality, and nutritional value. This ultimately causes huge economic losses in the agricultural and food industries. Excessive accumulation or production of melanin in humans also leads to cosmetic issues as well as serious skin diseases and disorders associated with hyperpigmentation, such as melasma, lentigo, age spots, and freckles.^3^ Overproduction and accumulation of melanin have also been linked to neurodegenerative diseases such as Alzheimer’s, Parkinson’s, and Huntington’s diseases. Therefore, the use of tyrosinase inhibitors is crucial in the food and agriculture industries for maintaining the quality of products, and in the medicine, cosmetics, and healthcare industries for the treatment of hyperpigmentation-related disorders, cancers, and skin lightening.^4^ Furthermore, melanin plays a critical role in protecting insects and arthropods through processes such as wound healing, defensive encapsulation, exoskeleton formation, and chitin synthesis. Thus, in addition to the application of tyrosinase inhibitors as additives in pharmaceutical, cosmetic, food, and beverage products, the use of these compounds is also important for controlling pests and harmful insect populations.^5^ The importance of controlling and eliminating pest insects in preserving agricultural products is very significant. Considering the importance of controlling and inhibiting tyrosinase activity in various industries, especially in the food and health sectors, extensive efforts have been made in recent years to discover, screen, design, and synthesize new tyrosinase inhibitors, with a particular focus on developing effective and safe compounds. In this regard, our main focus has also been on the investigation of antityrosinase activity of novel organic/inorganic synthetic or natural compounds.^6–12^ The objective of this study is also the assessment of the tyrosinase inhibitory potency of the essential oil isolated from the leaves of *Juniperus excelsa* subsp. *polycarpos* (also known as the Persian juniper) in an effort to find an appropriate, safe, and potent tyrosinase inhibitor. In recent years, plant extracts and essential oils, complex mixtures of volatile secondary metabolites-have attracted increasing interest in medical and cosmetic applications due to their better safety, wide range of biological activities, particularly in modulating inflammation and cancer-related processes, and also promising tyrosinase inhibitory potential. This trend is not necessarily a reflection of the inherent superiority of natural products over synthetic compounds in terms of structure or side effects, but rather arises from practical concerns, such as limited stability, suboptimal efficacy, or formulation challenges associated with certain synthetic compounds. For these reasons, there is great interest in developing natural medicinal products derived from plants that offer lower side effects, lower toxicity, better bioavailability, and greater safety compared to synthetic ones.^13^ When it comes to skin care products, herbal extracts and essential oils have many benefits, including their ability to inhibit tyrosinase and reduce hyperpigmentation disorders, as well as their antioxidant and antimicrobial activities.^14^ Chen et al. (2014) investigated the effect of apple polyphenols on tyrosinase and found that they have an inhibitory potency on the enzyme activity.^15^ Asghari et al. (2019) demonstrated the significant inhibitory effects of wild mint essential oil on tyrosinase activity.^16^ Menezes et al. (2020) also confirmed the presence of a strong tyrosinase inhibitory element in cinnamon essential oil.^17^ In this context and for the first time, the present study was also conducted to evaluate the inhibitory potential of the essential oil obtained from the leaves of a native juniper plant (*J. excelsa* subsp. *polycarpos*, Zanjan, Iran) on the activity of *Agaricus bisporus* tyrosinase, as a model enzyme for inhibition studies on tyrosinase. Our aim was to discover and introduce effective and safe compounds that can be used as preservatives and additives in food and cosmetic products.

*J. excelsa* subsp. *polycarpos* belongs to the Cupressaceae family and is characterized by blue and dark berries. It is found in countries, including Turkey, Syria, Lebanon, Georgia, Armenia, Azerbaijan, and Iran. In Iranian culture, the cypress has long been revered as a symbol of immortality. *J. excelsa* is a versatile medicinal herb and was historically employed for various ailments, such as jaundice, rheumatism, painful periods, wound recovery, cough, bronchitis, colds, tuberculosis, and hemorrhoids. The local people in Iran use it as a natural remedy for heart conditions, nervous disorders, etc.^18^

*J. excelsa* essential oil (JEO) exhibits a wide range of biological activities, in addition to tyrosinase inhibition. Its major constituents, such as α-pinene, α-cedrol, and limonene, which can influence oxidative stress, immune response, and cell proliferation, have also been shown to possess strong antibacterial, anti-inflammatory, antifungal, antileishmanial, and antioxidant properties.^19,20^ For instance, essential oil derived from both the leaves and berries has demonstrated significant inhibitory effects against pathogenic bacteria such as *Staphylococcus aureus* and *Escherichia coli*, as well as the fungus *Trichophyton rubrum* (MIC = 64–128 μg mL^–1^).^21,22^ In another study, it exhibited notably lower cytotoxicity compared to chlorhexidine, a commonly used antiseptic.^23^ Moreover, its antioxidant capacity, antiproliferative activity, and antileishmanial effects have been reported at nanogram-level concentrations (IC_50_ ≈ 0.0065–0.0093 μg mL^–1^), highlighting its strong therapeutic potential.^24,25^ These data highlight the importance of further exploring *J.* *excelsa* *oil* for its multifunctional therapeutic potential.

In the present study, indigenous juniper plants from the forests of the Tarom region in Zanjan were utilized. The obtained essential oil was analyzed and evaluated for its tyrosinase inhibitory potency. Enzymatic assays were performed using different amounts of essential oil using the dopachrome method. In all experiments, kojic acid served as a positive control and a standard inhibitor. The IC_50_ values were calculated, and the kinetic studies were conducted at different initial amounts of the essential oil to estimate the kinetic parameters and also the inhibition mode. The copper-chelating and antibrowning potencies of the essential oil were then investigated. Enzyme intrinsic fluorescence measurements were performed by using different amounts of essential oil in order to study the enzyme–essential oil interactions and determine the important binding parameters. Interactions between the enzyme and the most abundant compounds of essential oil were also theoretically investigated using the molecular docking approach. Finally, other biologically important activities of the essential oil, including antioxidant, antibacterial, and hemolytic activities, were also *in vitro* evaluated.

## 2 Materials and Methods

### 2.1 Chemicals

L-3,4-dihydroxyphenylalanine (L-DOPA), mushroom tyrosinase, kojic acid, and 1,1-diphenyl-2-picrylhydrazyl (DPPH) were obtained from Sigma-Aldrich Co. (St. Louis, MO). Other chemicals were obtained from Merck and Sigma-Aldrich. For all represented data, at least 3 independent experiments were carried out in triplicate, and the results represent standard experimental data.

### 2.2 Plant Materials and Extraction

At the end of the spring season in 2023, plant leaf materials were collected from the outskirts of Tarom (Zanjan province, Iran) and transported to the research laboratory. Subsequently, the separated portions of the samples were completely air-dried (away from sunlight) under safe conditions to avoid microbial and fungal contamination for several days. The crushed and divided leaves of *J. excelsa* were subjected to steam distillation for 4 h using a Clevenger-type apparatus. A bright yellow essential oil was then obtained. Hydrodistillation of 50 g of dried *J. excelsa* plant leaf materials yielded approximately 500 μL of essential oil, corresponding to a 1% (v/w) extraction yield. The oil was dried using dry sodium sulfate and stored in brown glass bottles and microtubes at 4 °C before analysis. In the calculations, the density of the *Juniperus* essential oil was approximated as 0.9 g mL^–1^ based on reported literature values.

### 2.3 Analysis Using Gas Chromatography Combined with Mass Spectrometry (GC-MS)

Gas chromatography–mass spectrometry (GC-MS) is one of the most powerful analytical techniques used for determining and measuring the chemical constituents in various samples. This allows a combination of the separation capabilities of gas chromatography with the detection and characterization capabilities of mass spectrometry. By utilizing two different methods, namely sample injection and thermal desorption, the GC-MS technique allows for the analysis of a broad range of volatile and semivolatile compounds in diverse matrices.^14^ After the extraction process, the obtained essential oil of *J. excelsa* subsp. *polycarpos* was analyzed using the GC-MS method in order to identify and quantify the chemical compounds present in the essential oil. For this purpose, about 1 mL of the essential oil was injected into the GC-MS instrument (Agilent 7890*B*/5977A).

### 2.4 Enzymatic Studies

#### 2.4.1 Mushroom Tyrosinase Assay

Diphenolase activity of mushroom tyrosinase was spectrophotometrically determined using L-DOPA as the substrate (final concentration of 2 mM) in different amounts of *J. excelsa* essential oil (JEO), according to our previously published protocol.^6,7^ Sodium phosphate buffer (100 mM, pH 6.8) was used for enzymatic assays, and the tests were conducted at room temperature using kojic acid as the standard inhibitor. The enzyme and substrate were also prepared in the same buffer, and JEO was prepared in dimethyl sulfoxide (DMSO). Different concentrations of the essential oil were prepared by diluting the JEO sample in phosphate buffer (final concentration range of 0.8 to 5.5 ppm). The final concentration of DMSO never exceeded 0.5% (v/v) in any of the assays, a concentration that had no effect on the measured parameters. Enzymatic assays were carried out after incubating the resulting mixtures with the enzyme for 15 min. The absorbance measurements were carried out for 10 min at 475 nm, and the slope values of the graphs of absorbance versus time (min) were considered as the measure of enzyme activity in each experiment. The enzyme inhibition values were finally calculated as described previously.^6,7^

#### 2.4.2 Kinetic Studies

To determine the type of enzyme inhibition and calculate the kinetic parameters, enzymatic assays were conducted at substrate concentrations of 0.5–3.0 mM (with intervals of 0.5 mM) using different amounts of the plant essential oil (final amounts of 0.2, 0.4, and 0.6 ppm of essential oil). The corresponding Michaelis–Menten, Lineweaver–Burk, and secondary plots were then drawn to estimate the kinetic parameters and inhibition constants.

### 2.5 Copper Ions Interaction Test

According to the literature, when some tyrosinase inhibitors (featuring distinctive absorption peaks) interact with Cu^2+^ ions present in the enzyme active center, the absorption peaks might show shifts.^15^ To explore the chelating properties of JEO toward copper ions, copper ion-containing solutions were prepared using phosphate buffer (100 mM, pH 6.8) and CuSO_4_·5H_2_O at different concentrations (final concentrations of 25, 50, and 100 μM). Then, these solutions were mixed with an equal volume of JEO and allowed to incubate at room temperature for 15 min. The absorption spectra were finally recorded between 240 to 700 nm using an ultraviolet spectrophotometer (Ultrospec 3100pro). Analysis of the obtained spectra can help us ascertain whether the essential oil interacts with the copper ions.^26^

### 2.6 Determining the Antibrowning Effect of JEO

To assess the antibrowning activity of JEO, fresh, undamaged fruits (banana and apple) and vegetables (potato) were obtained from the local market. They were washed with distilled water and cut into equal-sized pieces using a sharp knife. After cutting, the samples were treated with the desired amount of juniper essential oil (3.1 ppm). The samples treated with distilled water and kojic acid were used as negative and positive controls, respectively, in each test. Two grams of each sample were separately homogenized in 10 mL of 10% trichloroacetic acid (TCA) and 40 mL of distilled water to ensure homogeneity. The mixtures were then incubated at 37 °C for 2 h. Subsequently, they were filtered by using filter paper. The absorbance of the resulting samples was subsequently measured at a wavelength of 420 nm using a spectrophotometer (Ultrospec 3100pro). The proportion of melanin production was ultimately determined using the following formula (eq 1)­

1 $$\mathrm{MP}=\left[A_{a}/A_{c}\right]\times 100$$

where MP is the percentage of melanin production, and *A*_a_ and *A*_c_ represent the absorbance values (at 420 nm) for the sample and control, respectively.^27^ In addition to the quantitative tests, qualitative and visual analyses were also performed on the treated samples, and the corresponding images were obtained at given time intervals.

### 2.7 Ligand Binding Studies

For the investigation of protein–ligand interactions, both experimental (fluorescence spectroscopy) and theoretical (molecular docking) approaches were used as described below.

#### 2.7.1 Molecular Docking Studies

Molecular docking analyses were performed to anticipate the binding sites and interactions of the most abundant compounds of the essential oil (obtained from GC-MS analysis) and mushroom tyrosinase using the AutoDock 4.2 program. The three-dimensional (3D) structure of the enzyme (PDB ID: 2Y9X) was acquired from the Protein Data Bank, and additional molecules, including tropolone (as the ligand bound to the enzyme) and water molecules, were removed using ViewerLite software. The structures of the ligands bicyclo[3.1.1]­hept-2-ene,3,6,6-trimethyl, cedrol, and (1S,2E,6E,10R)-3,7,11,11-tetramethylbicyclo[8.1.0]­undeca-2,6-diene were drawn using the Chem3D program, and optimization was carried out using Gaussian 09W.v7.0 software. The ligands and enzymes were then brought together by using the Visual Molecular Dynamics (VMD) program. Subsequently, polar hydrogens and the prepared copper ion parameters were added to run AutoGrid. Docking was performed using the AutoDock 4.2 program with the Lamarckian genetic algorithm. Ligplot and Discovery Studio 2016 Client software were then used for analyzing the docking results and investigating the generated interactions.^28^

#### 2.7.2 Fluorescence Spectroscopy

A fluorescence spectrophotometer (Varian Cary Eclipse) was utilized to record the intrinsic fluorescence emission spectra of the enzyme, in the absence of essential oil, and with varying quantities of essential oil. The instrument parameters were set, as previously reported.^6,7^ Before the fluorescence measurements, the absorption and emission characteristics of the essential oil were separately investigated, and it was ensured that there was no interference with the enzyme emission. Measurements were performed at room temperature using a quartz fluorescence cuvette with a volume of 0.7 mL and a path length of 1 cm. The final concentration of the enzyme in the cuvette was 0.4 μM, and it was prepared in 100 mM sodium phosphate buffer, pH 6.8. The cuvette was then placed in the sample chamber, and the solutions of the essential oil (prepared in sodium phosphate buffer, following initial dissolution in DMSO) were repeatedly added to the cuvette in microliter volumes. The obtained intrinsic fluorescence intensity values were adjusted to prevent the influence of the internal fluorescence filtering effect.^29^ Analyzing the fluorescence quenching results helps to estimate the type of interactions that might occur between the fluorophore(s) and ligands. The fluorescence quenching data, including the binding constant (*K*_A_), Stern–Volmer constant (*Ksv)*, quenching constant (*k*_q_) values, and the number of binding sites (*n*), were finally calculated according to previously reported formulas.^6,7,29^

### 2.8 Evaluation of Other Biological Effects of JEO

#### 2.8.1 Measurement of Antioxidant Activity

To evaluate the antioxidant potency of the essential oil, the standard DPPH method was employed. This method is based on the ability to donate hydrogen and change the color of the ethanol solution of DPPH from purple to yellow (nonradical DPPH).^30^ For the antioxidant assay, the desired amount of plant essential oil was extracted, and the final concentrations of 1.53, 3.06, 6.11, and 9.17 ppm were mixed with an equal volume of DPPH solution (20 μM in ethanol) at room temperature. The incubation was performed in darkness (30 min), and the samples were then poured into the wells of a 96-well plate. Changes in absorbance values at 517 nm were recorded by using a spectrophotometer (Cytation5 imaging reader). Ascorbic acid was used as a positive control, and the tests were repeated at least three times. Finally, the percentage of DPPH radical scavenging activity was estimated using eq 2. This value indicates the antioxidant activity of the essential oil.

2 $$\mathrm{IP}=\left[A_{c}-A_{a}/A_{c}\right]\times 100$$

where IP is the inhibition percentage, and *A*_c_ and *A*_a_ are the absorbances of the negative control and sample, respectively.^31,32^

#### 2.8.2 Antibacterial Activity Assay

In this study, the *in vitro* antibacterial effects of JEO were investigated against the Gram-negative strain *E. coli* (PTCC 1330) and the Gram-positive strain *S. aureus* (PTCC 1112), utilizing a modified Kirby–Bauer disc-diffusion method.^33^ Each bacterial strain was initially cultured in a Luria–Bertani (LB) medium and incubated overnight at 37 °C. Subsequently, an appropriate volume of the culture was evenly distributed on sterile LB medium plates. Whatman paper discs (6 mm diameter) were then saturated with different volumes (20, 40, 60, 80, and 100 μL) of diluted JEO samples, which were prepared in sterile phosphate-buffered saline (PBS) and filtered (the final amounts of these volumes were almost equal to 140, 281, 421, 562, and 703 μg, respectively). The discs were uniformly placed on the agar plates and incubated at 37 °C for 19 h. Sterile water and discs containing tetracycline (30 μg) served as the negative and positive controls, respectively, throughout the experiment. Following the incubation period, the plates were taken out for evaluation of antibacterial activity.

#### 2.8.3 *In* Vitro Hemolysis Assay

For further assessment of the biological effects of JEO, an *in vitro* hemolysis assay was conducted. After obtaining a 2 mL blood sample from a healthy donor provided by a local blood bank, the sample was centrifuged at 3500 rpm for 5 min, and the resulting plasma was removed. The remaining red blood cells (RBCs) were suspended in an equal volume of PBS and centrifuged five times at the same speed. Next, RBC suspensions were prepared with a hematocrit of 1% and treated with various volumes of JEO (20, 40, 60, 80, and 100 μL) and incubated for 3 h in a water bath (37 °C). After incubation, the samples were centrifuged again (500 rpm, 10 min), and the absorbance of the supernatants was measured at a wavelength of 540 nm (Ultrospec 3100pro). These values are indicative of the amount of hemoglobin released from RBCs (hemolysis). The degree of hemolysis was calculated by comparing it to that of PBS and Triton X-100 as the negative and positive controls, respectively, using the following formula (eq 3):

3 $$h\mathrm{emolysis}\;\%=\left[\left(A_{S}-A_{\mathrm{NC}}\right)/\left(A_{\mathrm{PC}}-A_{\mathrm{NC}}\right)\right]\times 100$$

where *A*_s_ is the absorbance value of the treated JEO sample, *A*_NC_ and *A*_PC_ are, respectively, the absorbance values of the negative and positive controls (RBCs treated with PBS and Triton X-100, respectively). As in all other experiments, the tests were conducted at least in triplicate, and the calculations were based on the average values.^6,7^

## 3 Results and Discussion

### 3.1 Preparation of JEO and Identification of Its Chemical Constituents

To extract the essential oil, dried leaves of *J. excelsa* subsp. *polycarpos* were subjected to distillation. The chemical composition of the essential oil (EO) was analyzed using GC-MS (Supporting Information Figure S1), and the identified constituent compounds (42 constituents) along with their retention times are listed in Table 1.

**Table 1:** Chemical Constituents of the Essential Oil of *J. excelsa* subsp. *p*o*lycarpos* Obtained Using GC-MS Analysis^a^

**no**.	**compound**	**peak area** (%)	**RT**
1	tricyclo[2.2.1.0(2,6)]heptane,1,3,3-trimethyl	0.35	9.018
2	**bicyclo[3.1.1]hept-2-ene**,**3**,**6**,**6-trimethyl**	**34.31**	9.263
3	camphene	0.44	9.489
4	bicyclo[3.1.1]heptane, 6,6-dimethyl-2methylene-,(1S)-	1.27	9.934
5	β-myrcene	2.29	10.004
6	3-carene	2.9	10.424
7	o-cymene	0.48	10.615
8	D-limonene	2.27	10.697
9	γ-terpinene	0.54	11.124
10	cyclohexene, 3-methyl-6-(1-methylethylidene)-	1.13	11.582
11	trans-verbeno	0.73	12.415
12	bicyclo[2.2.1]heptan-2-ol, 1,7,7-trimethyl-, acetate, (1S-endo)-	0.51	14.228
13	cyclohexene, 4-ethenyl-4-methyl-3-(1methylethenyl)-1-(1-methylethyl)-, (3R-trans)-	0.74	14.87
14	hexanoic acid, hexyl ester	0.5	15.195
15	α-cubebene	0.4	15.392
16	cyclohexane, 1-ethenyl-1-methyl-2,4-bis(1-methylethenyl)-, [1S-(1.α.,2.β.,4.β.)]-	1.18	15.519
17	(3R,3aR,7R,8aS)-3,8,8-trimethyl-6methyleneoctahydro-1H-3a,7-methanoazulene	3.76	15.932
18	**(1S**,**2E**,**6E**,**10R)-3**,**7**,**11**,**11-tetramethylbicyclo[8.1.0]undeca-2**,**6-diene**	**6.41**	15.977
19	1H-3a,7-methanoazulene, octahydro-3,8,8-trimethyl-6-methylene-, [3R(3.α.,3a.β.,7.β.,8a.α.)]-	1.46	16.034
20	cis-thujopsene	0.77	16.117
21	1,4,7,-cycloundecatriene,1,5,9,9-tetramethyl-, Z,Z,Z	0.89	16.327
22	(1S,4aR,8aS)-1-Isopropyl-7-methyl-4-methylene-1,2,3,4,4a,5,6,8a-octahydronaphthalen	0.99	16.422
23	γ-muurolene	0.58	16.486
24	naphthalene,1,2,3,4,4a,5,6,8a-octahydro-7-methyl-4-methylene-1-(1-methylethyl)-, (1.α.,4a.β.,8a.α.)	0.48	16.53
25	(1R,2S,6S,7S,8S)-8-isopropyl-1-methyl-3-methylenetricyclo[4.4.0.02,7]decane-rel-	2	16.6
26	(1S,2E,6E,10R)-3,7,11,11-tetramethylbicyclo[8.1.0]undeca-2,6-diene	1.87	16.715
27	naphthalene,1,2,3,4,4a,5,6,8a-octahydro-4a,8-dimethyl-2-(1-methylethenyl)-, [2R(2.α.,4a.α.,8a.β.)]	0.42	16.753
28	α-cuprenene	0.36	16.829
29	cyclohexene,4-ethenyl-4-methyl-3-(1methylethenyl)-1-(1-methylethyl)-, (3R-trans)-	2.99	16.899
30	1-isopropyl-4,7-dimethyl-1,2,3,5,6,8a-hexahydronaphthalene	4.54	16.95
31	6-epi-shyobunol	0.44	17.039
32	α-muurolene	0.68	17.128
33	(4aR,8aS)-4a-methyl-1-methylene-7-(propan-2ylidene)decahydronaphthalene	0.49	17.179
34	cyclohexanemethanol,4-ethenyl.α.,.α.,4-trimethyl-3-(1-methylethenyl)-, [1R-(1.α.,3.α.,4.β.)]	1.25	17.224
35	(2E,4S,7E)-4-isopropyl-1,7-dimethylcyclodeca-2,7-dienol	1.7	17.58
36	(3R,3aR,5S,6R,7aR)-3,6,7,7- etramethyloctahydro-3a,6-ethanoinden-5-ol	1.38	17.853
37	**cedrol**	**12.42**	18.006
38	isospathulenol	0.39	18.063
39	τ-cadinol	1.55	18.184
40	α-cadinol	1.17	18.324
41	2-naphthalenemethanol,1,2,3,4,4a,5,6,8a-octahydro-.α.,.α.,4a,8-tetramethyl-, [2R-(2.α.,4a.α.,8a.β.)]-	0.4	18.369
42	kaur-16-ene,(8.β.,13.β.)	0.58	22.185

a The most dominant compounds are shown in bold; RT, retention time.

The data obtained from the GC-MS analysis indicated that the components found in the essential oil consist predominantly of monoterpenes, particularly pinenes, such as α-pinene. The three major constituents were

bicyclo­[3.1.1]­hept-2-ene, 3,6,6-trimethyl (34.31%), cedrol (12.42%), and (1S,2E,6E,10R)-3,7,11,11-tetramethylbicyclo[8.1.0] undeca-2,6-diene or bicyclogermacren (6.41%).

In a study conducted by Weli et al. (2014), the predominant compounds of the JEO included bicyclogermacrene (21.2%) and α-pinene (77.1%).^34^ In another study, the predominant compounds in the essential oil were α-pinene and phyllocladene with the abundances of 15 and 10%, respectively.^35^ In any case, the chemical composition of the essential oil depends on the environmental growth conditions, geographical location, soil composition, and water availability. Therefore, the major components of JEO from different regions may vary from one another.^36^

### 3.2 Enzymatic Studies

#### 3.2.1 Mushroom Tyrosinase Inhibitory Activity of JEO

The inhibitory effect of JEO on mushroom tyrosinase activity was assessed using L-DOPA substrate, according to the procedure outlined in section 2.4.1. As kojic acid is routinely known as a standard inhibitor of tyrosinase, we used it as a positive control in all experiments. Based on these results, JEO showed a notably stronger inhibitory effect at lower concentrations. For instance, at 3.1 ppm, JEO achieved 41.5% inhibition compared to only 27.6% by kojic acid (Figure 1). The IC_50_ value of JEO (4.26 ppm) was also significantly lower than that of kojic acid (6.1 ppm), indicating a greater inhibitory efficiency at lower doses. Although kojic acid exhibited higher inhibition percentages at elevated concentrations, the stronger performance of JEO at low doses highlights its potential as a natural and effective tyrosinase inhibitor. Notably, this inhibitory potency is stronger than that of several reported essential oils, such as *Ammoides pusilla* (IC_50_ = 17.6–29.6 ppm), *Thymus vulgaris* (∼12.8 ppm), and *Mentha spicata* (>10 ppm). These results suggest that JEO has considerable potential as a natural tyrosinase inhibitor.^37–39^

**Figure 1:**
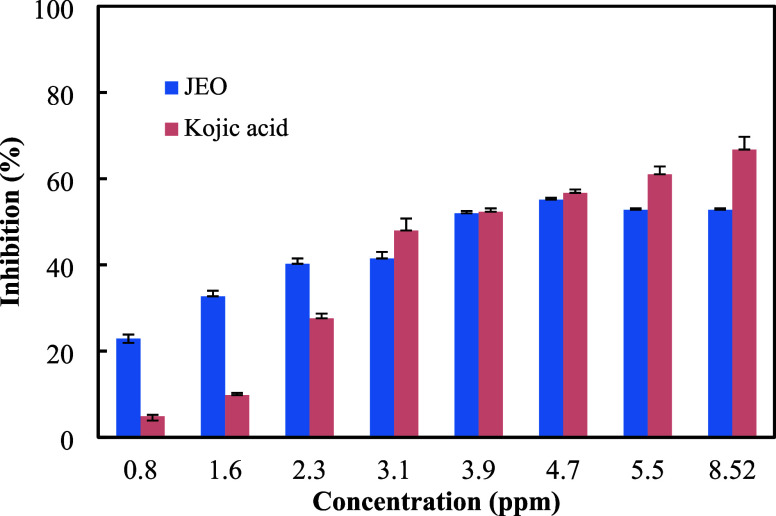
Percentage of enzyme inhibition in the presence of different concentrations of *J. excelsa* essential oil and kojic acid, as the standard inhibitor. The diphenolase activity of mushroom tyrosinase was assayed using the L-DOPA substrate. DMSO-dissolved essential oil samples were diluted with the assay buffer and preincubated with the enzyme (the final concentration of DMSO was below 0.5% and had no inhibitory effect on enzyme activity). L-DOPA was then added to the reaction mixtures, and the production of dopachrome was kinetically recorded at 475 nm. All assays were repeated at least 3 times (with 3 repeats each time), and the average values were considered in the calculations.

In the past, traditional medicine has employed various plant species to address skin pathologies and promote skin brightening effects. As a result, the use of herbal medicines has an ancient and rich history in several Asian countries. Recent reports and investigations have revealed that the utilization of plant-derived extracts has expanded in the production of cosmetics and personal care formulations. For example, in 2019, Moghrovyan and colleagues studied the impact of oregano essential oil on the activity of tyrosinase.

Their results indicated that terpenes are the main constituents of this essential oil, and the oil showed a significant inhibitory effect on mushroom tyrosinase activity.

^40^ The importance of hydroxyl groups in the inhibition of tyrosinase has been well highlighted in previous studies in terms of their molecular position, number, and specific interactions with the enzyme. Earlier studies have also identified that compounds possessing hydroxyl groups exhibit stronger inhibitory effects on tyrosinase. These groups interact with Cu^2+^ ions at the enzyme’s active site through coordination bonds, thereby chelating the metal ions and potentially participating in proton transfer during catalysis, and ultimately contributing to enzyme inactivation. Hydroxyl groups may also interact with the critical residues in the active site of the enzyme, leading to a decrease of the enzyme activity.^41^ Additionally, the presence of electronegative atoms, such as oxygen, in the structure of inhibitors increases their inhibitory potency.^42^ Based on the identified constituent compounds of JEO and comparison of our results with the previously reported data, it can be suggested that the tyrosinase inhibitory activity of JEO is likely due to the presence of various terpenic compounds and also alcohols in this essential oil (Table 1). However, there is insufficient information about intermolecular interactions between the enzyme and essential oil, since identifying the precise chemical substances responsible for the observed inhibitory effect would require further research due to the complex composition of the essential oil.

#### 3.2.2 Kinetic Study of Mushroom Tyrosinase Inhibition in the Presence of JEO

Enzyme kinetic analyses were conducted to determine the inhibition pattern of mushroom tyrosinase by JEO using Michaelis–Menten and Lineweaver–Burk plots. The obtained data were used for the estimation of the kinetic parameters of the enzyme (such as *K*_m_ and *V*_max_) in the absence and presence of different amounts of the essential oil (Figure 2A–D, Table 2). As shown in Table 2, the *V*_max_ value of the enzyme remained almost constant, with very slight alterations, while the *K_m_* value increased with increasing amounts of the essential oil. These results suggest that the inhibition type is mixed (competitive–noncompetitive), and the pattern lies between those for competitive and noncompetitive inhibition.

**Figure 2:**
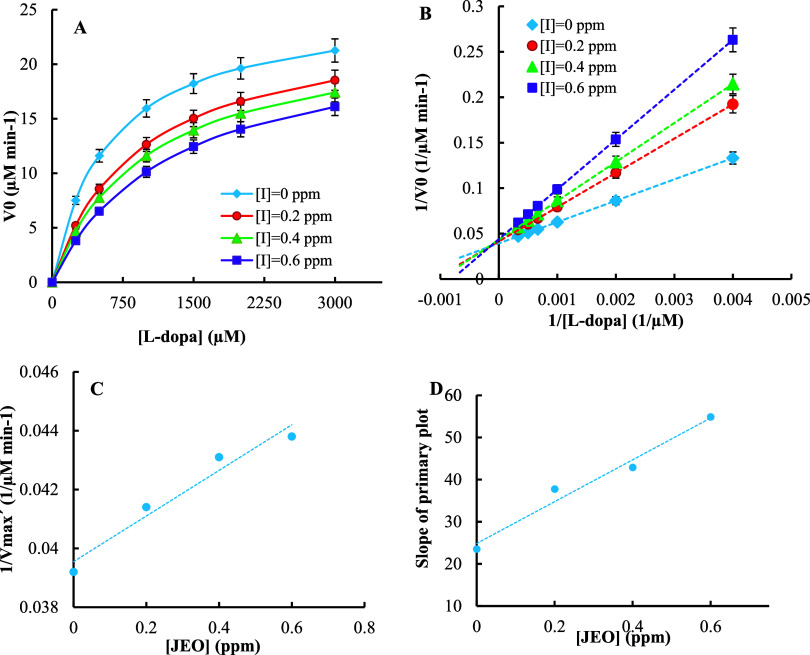
(A) Michaelis–Menten and (B) Lineweaver–Burk plots of the enzyme are presented, showing results both without and with different concentrations of *J. excelsa* essential oil over the concentration range of 0 to 2.0 mM of L-DOPA substrate. Secondary plots of 1/*V*_max_′ (C) and the slope of the primary plot (D) against various volumes of essential oil were used for the determination of the inhibition constants (*K*_I_ and *K_i_*, respectively).

**Table 2:** Kinetic Parameters and Inhibition Constants of Mushroom Tyrosinase in the Absence and Presence of Different Amounts of the *J. excelsa* Essential Oil

**constant**	
*K*_m_ (μM)	^a^602.40,^b^917.43,^c^1000.5,^d^1252.0
*V*_max_ (μM min^–1^)	^a^25.51,^b^24.15,^c^23.20,^d^21.75
*K*_I_ (μM)	5.14
*K_i_* (μM)	0.5
inhibition	reversible
inhibition type	mixed inhibition (competitive**–**noncompetitive)

a *K*_m_ and *V*_max_ values in the presence of 0 ppm essential oil.

b *K*_m_ and *V*_max_ values in the presence of 0.2 ppm essential oil.

c *K*_m_ and *V*_max_ values in the presence of 0.4 ppm essential oil.

d *K*_m_ and *V*_max_ values in the presence of 0.6 ppm essential oil.

In this situation, the plots cross to the left of the 1/V_0_ axis but above the 1/[S_0_] axis, and the active site as well as another site of the enzyme can bind to the inhibitor, or the active site binds to the inhibiting compound but does not hinder substrate binding.^43,44^ In this type of inhibition, the inhibition constant of the free enzyme (*K_i_*) is often significantly lower than that for the enzyme–substrate complex (*K*_I_), indicating a stronger inhibitor affinity toward the free enzyme. To determine these inhibition constants, secondary plots were also constructed by plotting 1/*V*_max_′ values against the various volumes of the essential oil ([I_0_], for calculation of *K*_I_) and slope of the primary plots against the various volumes of the essential oil ([I_0_], for calculation of *K_i_*) (Figure 2C,D).^43^ *K*_I_ and *K_i_* constants were 5.14 and 0.5 μM, respectively (Table 2).

Zhang et al. (2006) reported a competitive inhibition pattern for salicylic acid derivatives in the inhibition of *A. bisporus* tyrosinase based on Lineweaver–Burk plots and changes in *K*_m_ without significant alteration in *V*_max_.^45^ However, our kinetic analysis revealed a mixed-type inhibition pattern (competitive–noncompetitive) for the essential oil, indicating that the active components may interact with both the free enzyme and the enzyme–substrate complex. This difference may be attributed to the complex nature of the essential oil, which contains multiple bioactive constituents that could bind to diverse sites on the enzyme, unlike the single-compound inhibitors tested in Zhang et al.’s study.

To assess whether the inhibition of the enzyme by the essential oil (JEO) was reversible, a dialysis-based assay was performed. The enzyme–inhibitor mixture was dialyzed overnight against phosphate buffer (pH 6.8, 4  °C). After dialysis, a marked recovery of enzymatic activity was observed compared to the nondialyzed control, indicating that the inhibition was not due to irreversible covalent modification (Table 2). These findings suggest that the inhibitory effect of JEO on tyrosinase is reversible, supporting the hypothesis of a noncovalent binding mechanism.

### 3.3 Cu^2+^ Chelation Activity of JEO

Tyrosinases have two copper ions (Cu^2+^) in their active centers, which are crucial for enzyme activity. Many tyrosinase-inhibiting compounds exhibit natural chelating ability against these ions and finally result in potent inhibitory effects on tyrosinase activity.^46^ For evaluation of the possible copper ion chelating activity of JEO, measurements were performed by using the spectrophotometric method, as explained in section 2.5. The results revealed that upon the addition of copper ion solutions to the sample, the peak intensity decreased, suggesting that JEO might interact with copper ions, and one of the possible mechanisms for the observed inhibitory potency of the essential oil may result from this process (Supporting Information Figure S2). This finding aligns with previous studies that have confirmed the effects of some essential oils on metal ion chelation and tyrosinase inhibition activities. According to the literature, the interaction between chelating agents and copper ions can lead to changes in the absorption spectra, including variations in peak intensities, shifts in absorption maxima (either red or blue shifts), and sometimes the appearance of new absorption bands. The nature and extent of these spectral changes depend on the coordination environment and the structural properties of the ligand, which in turn reflect the formation and stability of Cu-ligand complexes.^47^

### 3.4 Antibrowning Effects of JEO

Browning is the most significant physiological damage that alters the visual quality and taste of agricultural products during harvesting, mechanical handling, and storage, resulting in decreased consumer acceptance. Tyrosinase is an essential enzyme that plays a crucial role in the browning process, and finding suitable ways to inhibit its activity would prevent or decrease the browning of agricultural products and food.^48^ Following the *in vitro* enzymatic studies, we also evaluated the antibrowning effects of the essential oil on some fruits and vegetables. Figure 3A shows the percentage of melanin production in fruit and vegetable samples treated with JEO compared to that of kojic acid. Melanin production in JEO-treated apple, banana, and potato samples was 49, 54, and 64%, respectively, while those treated with kojic acid were 63, 67, and 78%, respectively. Therefore, it emerges that the production of melanin decreased in the samples treated with essential oil compared to that with kojic acid. The observation that melanin production in treated apples was lower than that in both bananas and potatoes indicates that the essential oil (JEO) had a strong antibrowning effect. Despite apples naturally having higher tyrosinase activity, which typically makes them more prone to browning, JEO treatment significantly reduced melanin production in apples (49%), even lower than that in potatoes (64%) and bananas (54%). This suggests that JEO effectively inhibited tyrosinase activity in apples, which are usually more susceptible to browning. These results align with previous studies that highlight apples’ higher tyrosinase levels, making them more vulnerable to browning. While potatoes have lower tyrosinase activity, their browning is more influenced by polyphenol oxidase (PPO), which may also contribute to the observed effects. In summary, the varying antibrowning effects across the three samples reflect the differences in their endogenous enzymes, with JEO demonstrating strong potential as a natural and effective antibrowning agent, particularly for apple varieties that are typically more prone to browning. Additionally, comparative images of the fruit and vegetable samples after treatment are shown in Figure 3B and confirm the data obtained from the quantitative assays. Quantitative color analyses of these images using ImageJ software are shown in Supporting Information Figure S3. We also extended our antibrowning assay to evaluate the stability of the treated samples over longer periods, specifically 24 and 48 h. The results showed that the antibrowning effects of the essential oil (JEO) were sustained over time. Specifically, the inhibition of melanin production remained almost consistent for up to 48 h, with no significant increase in browning observed after the initial 2 h of treatment. The concentration of JEO used in the antibrowning assays was approximately 3.1 ppm, as discussed in section 2.6, which is notably lower than the typical concentrations of common synthetic preservatives such as sodium metabisulfite and ascorbic acid, which are generally applied in the range of 100–1000 ppm in food industry settings.^49^ Essential oils like JEO exhibit effective antibrowning effects at low concentrations (1–10 ppm), likely due to their bioactive compounds, providing advantages including lower toxicity and higher consumer acceptance.^50,51^ These findings suggest that JEO could serve as an efficient and safer natural alternative to conventional preservatives in food preservation. The results suggest that JEO could reduce melanin production in the treated samples and may be considered as a suitable candidate for further studies on decreasing tyrosinase-induced browning in fruits and vegetables.

**Figure 3:**
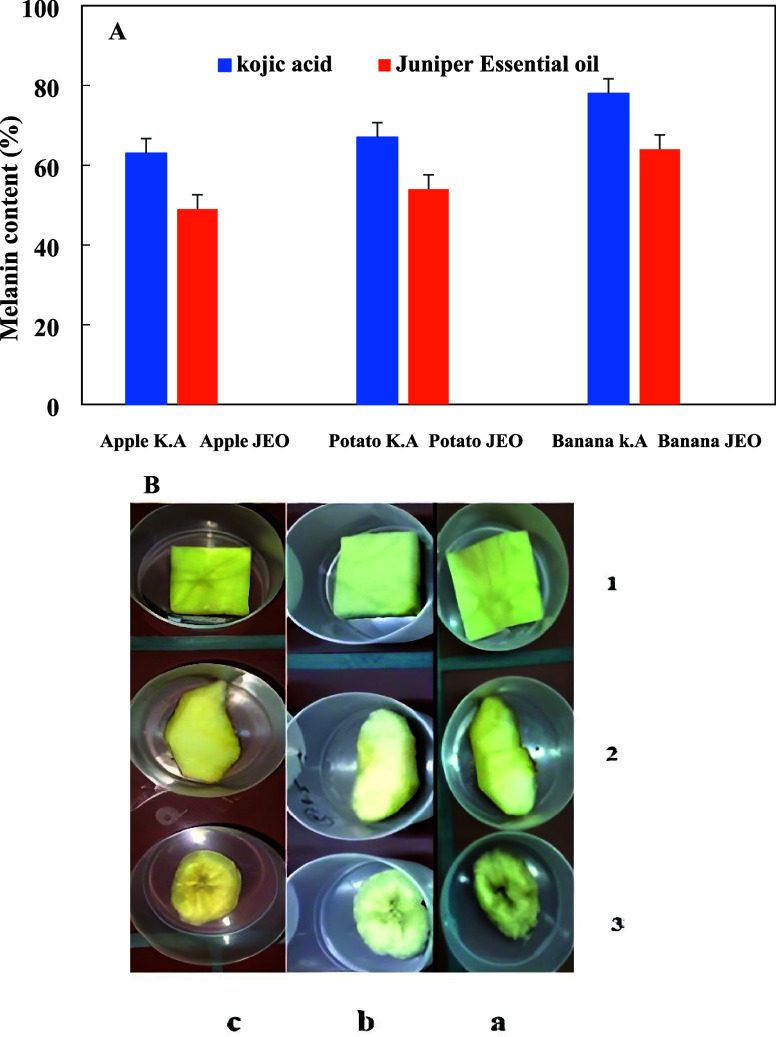
(A) Comparison of antibrowning activity of *J. excelsa* essential oil (JEO) and kojic acid (K.A) on fresh-cut fruits (banana and apple) and vegetables (potato). (B) Images of the antibrowning effects of JEO (∼3.1 ppm) on potato (row 1), apple (row 2), and banana (row 3). (a)–(c) Samples treated with distilled water (negative control), *J. excelsa* essential oil, and kojic acid (positive control), respectively.

### 3.5 Study of the Enzyme–Ligand Interactions

#### 3.5.1 Molecular Docking Analysis

To predict the types and sites of interactions between the enzyme and components of the essential oil, molecular docking studies were performed using AutoDock 4.2. For this end, the analyses were conducted between mushroom tyrosinase (PDB ID: 2Y9X) and the most dominant constituents of the essential oil (bicyclo[3.1.1]­hept-2-ene, 3,6,6-trimethyl, cedrol, and (1S,2E,6E,10R)-3,7,11,11-tetramethylbicyclo[8.1.0]­undeca-2,6-diene).^52^ The most stable complexes of the enzyme–ligand were selected for each ligand after running the docking simulations. Discovery Studio and LigPlot were then employed to visualize and analyze the interactions. Binding energies were calculated as −4.6, −7.4, and −6.1 kcal/mol for compounds bicyclo[3.1.1]­hept-2-ene, 3,6,6-trimethyl, cedrol, and (1S,2E,6E,10R)-3,7,11,11-tetramethylbicyclo[8.1.0]­undeca-2,6-diene, respectively. The binding sites and involved residues are shown in Figures 4 and S4, and detailed information on the interactions and bond distances is shown in Supporting Information Tables S1–S3. Based on the results, while none of the desired ligands interact directly with the copper-coordinating histidine residues, they exhibit various interactions with the key residues of the enzyme and bind to the sites previously identified as binding sites for potent inhibitors.^28^ The compound bicyclo[3.1.1]­hept-2-ene,3,6,6-trimethyl forms hydrophobic interactions (alkyl, π-alkyl) with the residues Pro329, Ile328, Ala323, Phe105, Tyr78, and Met325 (Figure 4A). According to the literature, some of the enzyme-inhibiting compounds, such as 4-bromophenol, tannins, and kushesenol A, are also involved in the interactions with the mentioned binding sites.^7,28,53^

**Figure 4:**
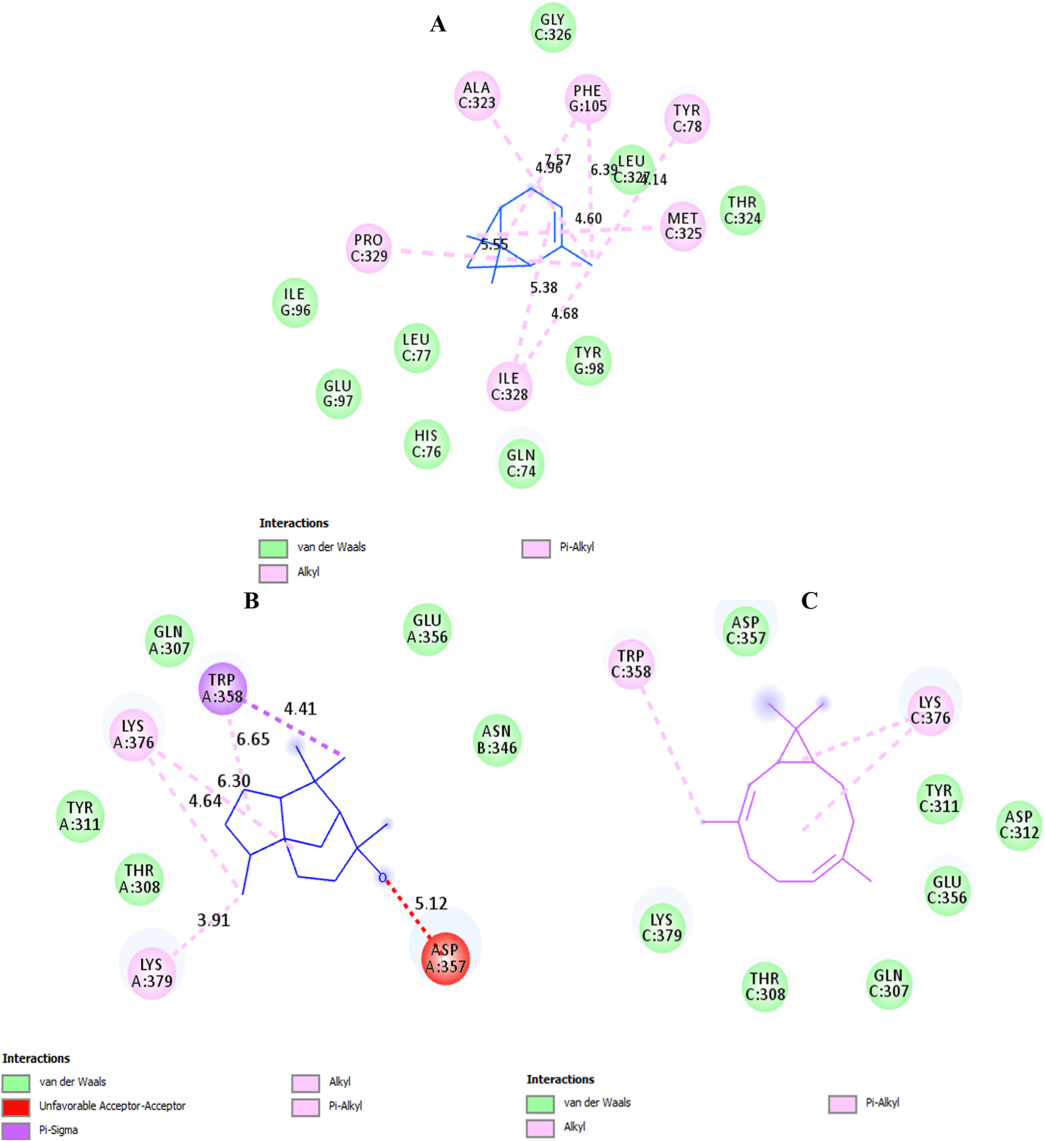
Results of molecular docking analyses of the predominant components of the essential oil (bicyclo[3.1.1]­hept-2-ene,3,6,6-trimethyl (A), cedrol (B), and (1S,2E,6E,10R)-3,7,11,11-tetramethylbicyclo [8.1.0] undeca-2,6-diene) (C) with mushroom tyrosinase. The images represent the results analyzed in Discovery Studio for each ligand.

Cedrol participates in hydrophobic interactions with residues Lys376, Lys379, and Trp358 (alkyl, π-alkyl, and π-sigma). Additionally, it forms interactions with Asp357, the nature of which may vary depending on the protonation state of this residue, potentially influenced by ligand binding (Figure 4B). It has been shown that Lys379 is an important residue for the binding of the substrate/ligand to mushroom tyrosinase.^53^ The best configuration of the docked compound (1S,2E,6E,10R)-3,7,11,11-tetramethylbicyclo[8.1.0] undeca-2,6-diene (Bicyclogermacrene) (Figure 4C) engages in hydrophobic interactions (alkyl, π-alkyl) with residues Trp358 and Lys376. A four-helical bundle (helices R3, R4, R10, and R11) is located at the center of tyrosinase and forms the catalytically vital binuclear Cu^2+^ binding site. Adjacent to the core region is a 27-residue extension that includes a short 3_10_-helix (residues 368 to 372) and an α-helix (α13, residues 375 to 391). This region is very essential for tyrosinase function.^54^

#### 3.5.2 Intrinsic Fluorescence Studies

Intrinsic fluorescence measurements were used to further study the interactions between the enzyme and essential oil. This technique is routinely used for studying the binding affinity between the enzyme and inhibitors.^29^ The obtained results can show whether the addition of the essential oil would result in the quenching of the enzyme fluorescence. Meanwhile, this may help us gain general information about the impact of essential oil compounds on the 3D structures of the enzyme. Since mushroom tyrosinase contains both Trp and Tyr residues in its structure, intrinsic fluorescence studies were carried out at excitation wavelengths of 280 and 295 nm. Figure 5A shows the fluorescence spectrum of the enzyme, which has strong emission at a wavelength of 340 nm, with no interference from the essential oil sample. With the addition of the essential oil to the enzyme solution, the enzyme fluorescence intensity decreased progressively at both excitation wavelengths (Figure 5A). The decrease of the intensity is not accompanied by obvious changes in the position of the emission peak at 280 nm, suggesting that no dramatic conformational changes occurred upon the binding of the essential oil components to the enzyme. It is important to emphasize that data obtained from fluorescence studies are generally indicative of local structural changes around the fluorophore residue(s) and are routinely extrapolated to the entire 3D structure of proteins.^29^

**Figure 5:**
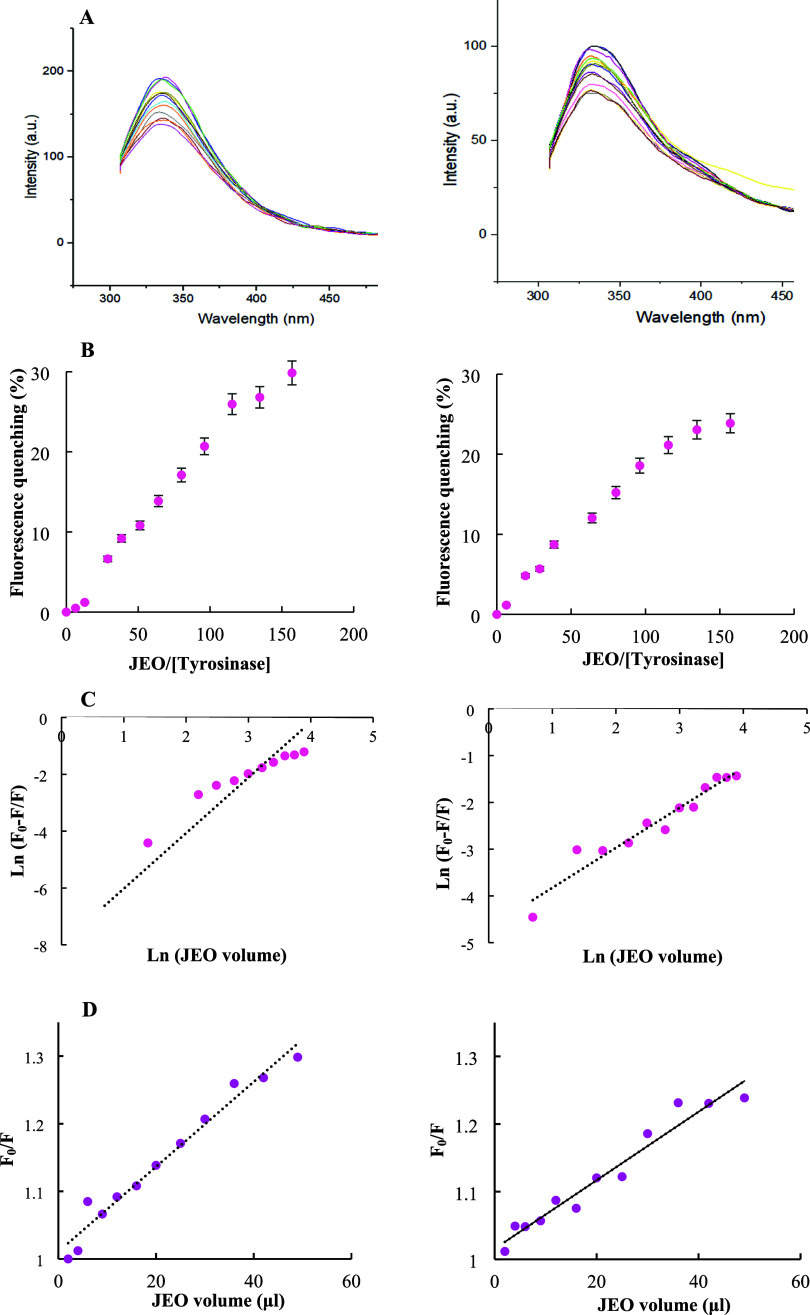
(A) Intrinsic fluorescence spectra of the enzyme at excitation wavelengths of 280 nm (left) and 295 nm (right) in the absence and presence of increasing amounts of essential oil (JEO). Enzyme and essential oil were prepared as described in the “Materials and methods” section. Measurements were carried out at room temperature with a final concentration of 0.4 μM of the enzyme. (B) Fluorescence quenching percentage of JEO as a function of the enzyme molar ratio at excitation wavelengths of 280 nm (left) and 295 nm (right). (C) and (D) Curves of Ln (F_0_-F/F) versus Ln [ligand] and Stern–Volmer plots at both excitation wavelengths (280 and 295 nm), respectively.

The percentage of fluorescence quenching of mushroom tyrosinase by essential oils was also quantitatively evaluated. For this end, the plots of fluorescence quenching percentage were depicted versus the ratio of the essential oil volume to the enzyme molar at both excitation wavelengths of 280 and 295 nm (Figure 5B). According to the results, the maximum fluorescence quenching values were ∼30 and 24% for the samples excited at 280 and 295 nm, respectively. Since the obtained maximum quenching values are close at both excitation wavelengths, it seems that the structural alterations induced by the essential oil components around the microenvironments of Trp residues are more obvious than those of Tyr residues. The greater number of Trp residues in the enzyme structure compared to Tyr can also justify this data.

The Stern–Volmer equation (eq 4) was employed for calculation of the Stern–Volmer (*K*_SV_) and bimolecular quenching rate (*k*_q_) constants as follows:

4 $$\frac{F_{0}}{F}=1+K_{\mathrm{SV}}\left[Q\right]=1+K_{q}\tau \left[Q\right]$$

where *F*_0_ and *F* represent the intensities of fluorescence emission in the absence and presence of the quencher, respectively, *K*_SV_ is the Stern–Volmer constant that reflects the strength of the quencher–fluorophore interaction, [*Q*] is concentration of quencher, *kq* is the bimolecular quenching rate constant in the presence of quencher, and τ is the average lifetime of the fluorophore in the absence of a quencher (τ = 10^–8^ s), respectively. As shown in Table 3, the estimated *K*_SV_ values were found to have the same order of magnitude at both excitation wavelengths (280 and 295 nm). The Stern–Volmer plots (especially at an excitation wavelength of 280 nm) are linear in the studied concentration range. One interpretation of this finding is that one class of fluorophore molecules is accessible to the quencher molecules.^29^ The calculated bimolecular quenching constants (*k*_q_) at both excitation wavelengths (280 and 295 nm) are below the limiting diffusion rate constants of the biomolecules with different quenchers (2 × 10^10^ M s^–1^) (Table 3). This suggests that the essential oil mainly quenches the enzyme fluorescence by a dynamic mechanism rather than a static one.^29^

**Table 3:** Binding Parameters for the Interactions of Essential Oil with the Enzyme^a^

**wavelength** (nm)	***K***_**SV**_ **(M** ^**1**^ **)**	***k*** **q** **(M** ^–1^ **s** ^–1^ **)**	***K***_**A**_ **(M** ^–1^ **)**	***n***
280	(6.2 ± 0.06) × 10^3^	(6.23 ± 0.06) × 10^5^	(3.4 ± 0.71) × 10^4^	1.13 ± 0.02
295	(5.10 ± 0.10) × 10^3^	(5.12 ± 0.10) × 10^5^	(9.2 ± 1.31) × 10^3^	0.85 ± 0.02

a These values were extracted from the Stern–Volmer and double logarithmic plots and include the Stern–Volmer constant (*K*_SV_), bimolecular quenching constant (*k*_q_), binding constant (*K*_A_), and binding stoichiometry (*n*).

Finally, the double logarithmic Stern–Volmer equation (eq 5) was used to calculate the number of binding sites for the ligand molecules (*n*) and the apparent binding constant (*K*_A_) (Table 3).^6,7^ For this end, the corresponding double logarithmic Stern–Volmer plots are depicted (Figure 5C). The slope and intercept of these graphs indicate the *n* and log *K*_A_ values, respectively (Table 3).

5 $$\ln\;\left(\frac{F_{0}-F}{F}\right)=n\;\ln\left[Q\right]+\log K_{A}$$

Considering the calculated *n* values (Table 3), the stoichiometry of the binding is 1:1, indicating the presence of only one binding site on the enzyme for the ligand molecules. The calculated *K*_A_ values (3.4 × 10^4^ and 9.2 × 10^3^) are also shown in Table 3.

### 3.6 Assessment of Other Important Biological Effects of the Essential Oil

#### 3.6.1 Antibacterial Effects

Multidrug-resistant organisms are one of the significant health issues around the world, and the situation is far from being under control. Therefore, efforts to find antibacterial compounds of natural origin have become a vivid area of research. Essential oils have potent bioactivities as antibacterial, antioxidant, insecticidal, and antifungal effects.^55^ The antibacterial susceptibility of JEO was tested using the modified Kirby–Bauer disk diffusion method against *S. aureus* (as the Gram-positive strain) and *E. coli* (as the Gram-negative strain) based on the formation of inhibitory zones (or the areas of clearance). In general, the larger diameter of the inhibition zone is indicative of the stronger antibacterial effect.^56^ According to the data (Figure 6 and Supporting Information Table S4), the essential oil showed an inhibitory effect against the Gram-positive (*S. aureus*) strain at volumes of 80 and 100 μL (almost equal to 562 and 703 μg), but it could not inhibit the growth of the Gram-negative (*E. coli*) one at the studied volume range (Figure 6). The areas of clearance against *S. aureus* were 18 and 21 mm for the final volumes of 80 and 100 μL (almost equal to 562 and 703 μg) of the essential oil, respectively (Supporting Information Table S4). In comparison, the standard antibiotic used as a positive control (tetracycline) exhibited a larger inhibition zone of 28 mm at a lower concentration (30 μg) under the same conditions. Although JEO demonstrated notable antimicrobial activity, its potency was lower than that of the standard antibiotic. Nonetheless, considering its natural origin and potential for lower side effects, JEO represents a promising alternative or complementary antimicrobial agent. Further studies are warranted to explore its full spectrum and mechanisms of action.

**Figure 6:**
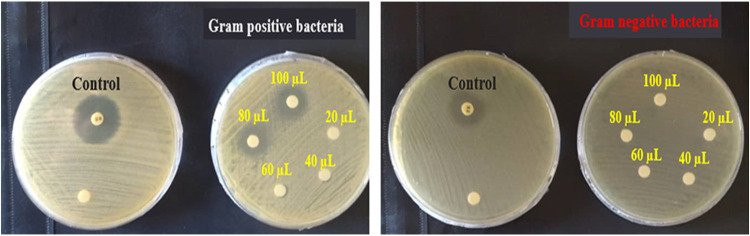
Inhibition zone (in mm) of JEO and control samples against Gram-positive (*S. aureus*, left) and Gram-negative (*E. coli*, right) bacteria. Discs of tetracycline (30 μg) and sterile water were utilized as the positive and negative controls, respectively. Volumes of 20–100 μL of the essential oil are almost equal to 140, 281, 421, 562, and 703 μg, respectively.

According to the literature, Gram-positive bacteria are generally more sensitive to antibacterial components than Gram-negative ones. The permeable cell walls of these bacteria do not usually restrict the penetration of antimicrobial compounds, while the outer membrane and sets of multidrug resistance pumps in Gram-negative bacteria are very efficient barriers to antimicrobials. Our data were also in good accordance with previous reports.^57^

The importance of identifying components of the essential oils and their correlation with antibacterial effects has been shown in previous studies. For example, it has been indicated that the presence of hydroxyl group-containing compounds greatly increases the activity of essential oils against multiple microorganisms.^58^ Furthermore, it is suggested that the antibacterial activity of the essential oil of *J. excelsa* might be attributed to the presence of bioactive compounds such as hydroxylated compounds and pinenes, which are the dominant components of the essential oil.^59^ As mentioned before, pinenes, such as α-pinene, are the predominant compounds in our essential oil (Table 1).

#### 3.6.2 Antioxidant Activity

Plant essential oils and extracts have attracted growing attention as natural antioxidant compounds due to their relatively less damaging effects on human and animal health and the environment. This makes them suitable candidates for application, especially in the food and pharmaceutical industries.^60^ For further biological characterization of the obtained essential oil and considering the growing demand for developing natural antioxidants, the antioxidant potency of JEO was evaluated using DPPH at different concentrations, as mentioned in the “Materials and Methods” section, and ascorbic acid was used as the positive control in all tests (Figure 7). Comparative analysis with ascorbic acid, a standard antioxidant (3.5 ppm), revealed that JEO at a slightly lower concentration (3.06 ppm) exhibited notably higher scavenging activity (∼139 vs 100%). This suggests that JEO may possess potent antioxidant properties that surpass those of ascorbic acid. Moreover, a clear dose-dependent enhancement in radical scavenging was observed with increasing JEO concentrations. For better visualization, an inset chart comparing JEO and ascorbic acid at equivalent concentrations is embedded within the main graph (Figure 7).

**Figure 7:**
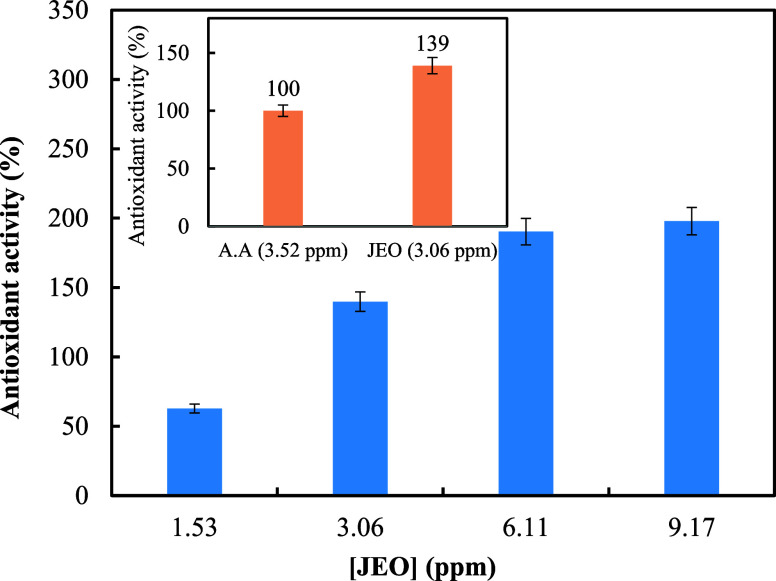
The free radical scavenging ability at varying concentrations of *J. excelsa* essential oil was assessed using the DPPH method. The inset chart compares JEO and ascorbic acid (A.A., as a positive control) at almost equivalent concentrations. The data presented are average values derived from a minimum of three independent experiments.

Previous reports have also shown that the essential oils obtained from juniper plants exhibit antioxidant properties.^26^ For instance, in the study conducted by Zheljazkov and colleagues, the antioxidant activities of several juniper species were compared (including *Juniperus communis*, *Juniperus sibirica*, *J. excelsa*), and the results indicated that the antioxidant potential of *J. sibirica* was higher than that of the other two species. Furthermore, *J. excelsa* and *J. communis* were found to have almost the same antioxidant capacity.^61^ In another study, Al-Busafi et al. suggested that the antioxidant properties of essential oils extracted from different parts of *J. excelsa* might be generally due to the interaction of their components, especially terpenes, with DPPH and their ability to donate hydrogen to this free radical.^27^ In line with the previous data, the presence of monoterpenes, as the most abundant components of our obtained JEO, could be accounted for as one of the determinant factors for the observed antioxidant potency. The phytochemical diversity of JEO (Table 1) may also be responsible for the antioxidant effect, and both major and minor components may work synergistically together. Bartikova et al. reported that the DPPH scavenging activity of *Piper betle* essential oil was correlated with the amounts of monoterpenes and sesquiterpenes present in the plant essential oil.^46^

#### 3.6.3 Hemolytic Activity

The hemolytic potential of natural products or drugs can induce various mechanisms, such as increased membrane permeability of red blood cells, leading to their lysis. Thus, hemolysis is considered as a fundamental problem for some biological substances. *In vitro* hemolytic assays are routinely used to investigate the damage caused by essential oil components to RBC membranes.^62^ The hemolytic activity of JEO was investigated according to the procedure described (section 2.10.3). The results revealed that the integrity of the erythrocyte membranes was maintained in the tested range, and these membranes were impermeable to the essential oil (Supporting Information Figure S5). The maximum hemolysis induced by the essential oil was 3.6% (at 100 μL, ∼8 ppm), remaining below the 5% threshold defined as nonhemolytic by ASTM E2524-08,^63,64^ indicating acceptable hemocompatibility. We also observed no significant cell aggregation, indicating that JEO has no hemolytic effect and can be considered safe at the tested range. This standard has also been supported by recent literature emphasizing that plant-derived compounds or nanomaterials exhibiting hemolysis below 5% are considered hemocompatible.^65^

## 4 Conclusion

Considering the importance of effective and safe tyrosinase inhibitors in preventing unwanted browning of vegetables and fruits, and treating skin cancers and disorders, and melanin accumulation-related conditions, the introduction of natural antityrosinase compounds is highly demanded in various areas. Effective components in natural resources (e.g., plants) have good potential for tyrosinase inhibition. In this regard, we evaluated the inhibitory effects of the essential oil of *J. excelsa* subsp. *polycarpos*, which naturally grows in Iran, on mushroom tyrosinase for the first time. This species is one of the native medicinal plants and is widely used by local people for the traditional treatment of various diseases. GC-MS analyses revealed that the obtained essential oil is rich in monoterpenes, particularly pinenes, and the compounds bicyclo[3.1.1]­hept-2-ene, 3,6,6-trimethyl, cedrol, and (1S,2E,6E,10R)-3,7,11,11-tetramethylbicyclo[8.1.0]­undeca-2,6-diene (Bicyclogermacrene) are the major constituents of the essential oil. Although, according to the observed phytochemical diversity of the essential oil components, it can be suggested that both major and minor components might work together in synergistic ways to manifest the essential oil’s biological activities. The enzymatic assay results showed that this essential oil displays a moderate to good inhibitory effect on diphenolase activity of the enzyme at the tested range. Based on the kinetic analysis, it emerges that the inhibition mode of the essential oil was mixed (competitive–noncompetitive). Copper chelation assay suggested that the essential oil components might interact with the active center copper ions, and one of the possible mechanisms for the observed inhibitory effect may result from this process. Ligand binding data showed that all components of the essential oil are involved in different interactions with the important residues of the enzyme and dynamically quench its intrinsic fluorescence in a stoichiometric ratio 1:1. The essential oil also showed a good antibrowning effect on the tested vegetables and fruits, and may decrease tyrosinase-induced hyperpigmentation-related problems in the food and agriculture industries. Further biological characterization of the essential oil revealed that it also has antimicrobial and antioxidant properties and no hemolytic effects. These features open up the possibility of its applications in different research and industrial areas. However, before any potential application, more studies on its other bioactivities and toxicity are warranted.

## Supplementary Material



## Notes

GC-MS chromatogram of the essential oil obtained from the leaf materials of *J. excelsa* subsp. polycarpos (JEO); UV spectrum of JEO alone or with different concentrations of Cu^2+^ to investigate copper-chelating activity of JEO; results of molecular docking analyses of the predominant components of JEO with the enzyme; details of interactions between mushroom tyrosinase and predominant components of JEO; effect of JEO on the growth of Gram-positive and Gram-negative bacteria; *in vitro* hemolytic effects of different concentrations of JEO on human red blood cells (PDF)
