# Supplementary material for: Insights into the Mushroom Tyrosinase Inhibitory, Antibrowning, Antioxidant, and Other Biologically Important Actions of Juniperus excelsa subsp. polycarpos (Persian Juniper) Essential Oil: In Vitro and In Silico Studies
Source: ACS Omega. 2025 Nov 25;10(48):58441–55. doi: 10.1021/acsomega.5c06131 (PMC13103980; doi:10.1021/acsomega.5c06131)
Supplement: Supplementary file 1 [file ao5c06131_si_001.pdf]

Supporting information

For

Insights into Mushroom Tyrosinase Inhibitory, Anti-browning, Antioxidant and Other Biologically Important Actions of *Juniperus excelsa* subsp. *polycarpos* (Persian Juniper) Essential Oil: *In vitro* and *In silico* studies

Atiyeh Mahdavi [a,*], Mehdi Zanghaneh [a], Parviz Moradi [b, c*]

[a]Department of Biological Sciences, Institute for Advanced Studies in Basic Sciences (IASBS), 444 Prof. Sobouti Blvd., Gava Zang, Zanjan 45137-66731, Iran.

[b]Julius Kühn Institute (JKI) – Federal Research Centre for Cultivated Plants, Institute for Breeding Research on Horticultural Crops, Erwin-Baur-Str. 27, D-06484 Quedlinburg, Germany

[c]Zanjan Agricultural and Natural Resources Research & Education Centre, AREEO, Zanjan 45195, Iran

***Corresponding Authors:**

Atiyeh Mahdavi, Ph.D. Department of Biological Sciences, Institute for Advanced Studies in Basic Sciences (IASBS), P.O. Box 45195-1159, Zanjan, Iran. Fax: +98 2433155142. E-mail: a.mahdavi@iasbs.ac.ir, Atiyeh.Mahdavi@gamil.com.

Parviz Moradi, Ph.D. Julius Kühn Institute (JKI) – Federal Research Centre for Cultivated Plants, Institute for Breeding Research on Horticultural Crops, Erwin-Baur-Str. 27, D-06484 Quedlinburg, Germany. E-mail: Parviz.Moradi@julius-kuehn.de.

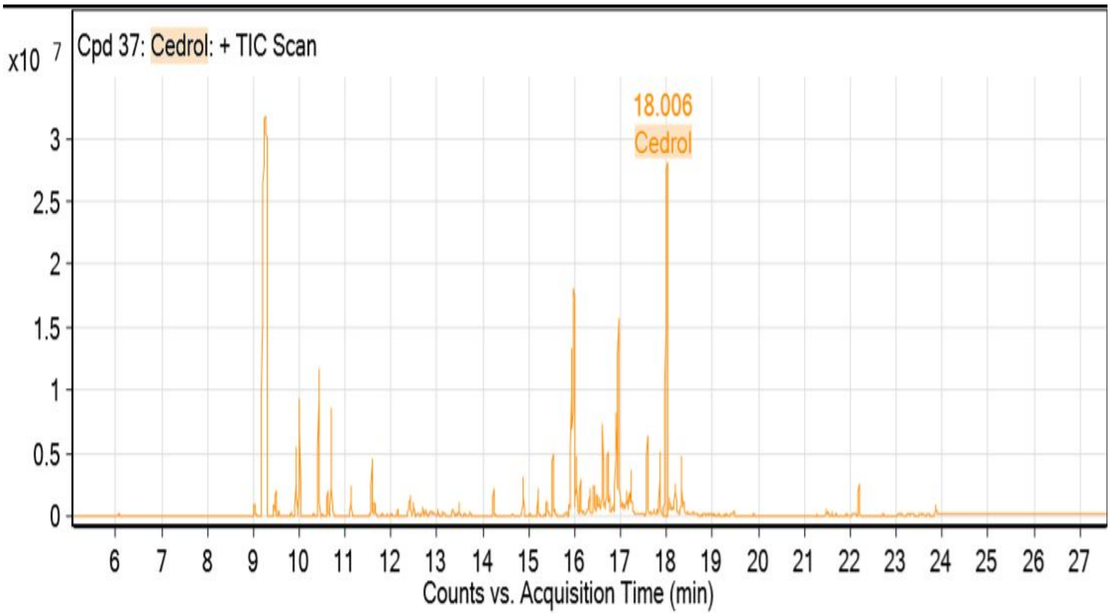

Fig. S1. The GC-MS chromatogram of the essential oil obtained from plant leaf materials of *J. excelsa* subsp. polycarpos.

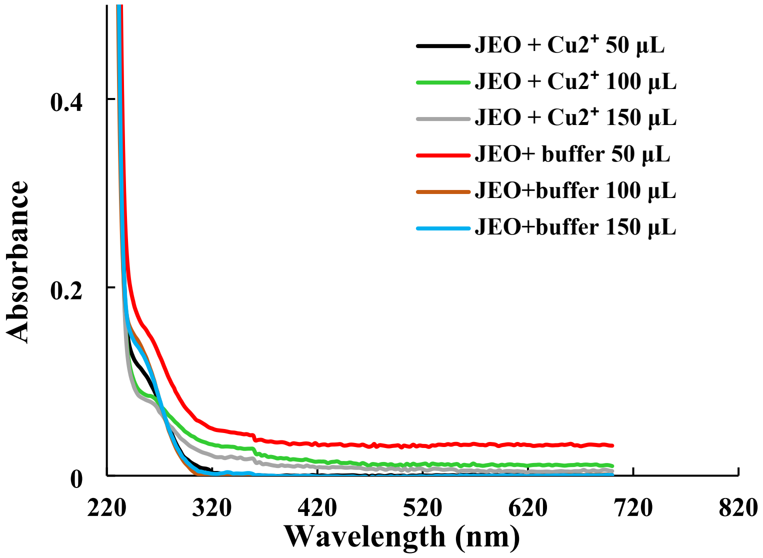

Fig. S2. The UV spectrum of the essential oil alone or with different concentrations of Cu^{+2} to investigate copper-chelating activity of the essential oil. Copper ion-containing solutions were prepared using phosphate buffer (100 mM, pH 6.8) and $CuSO_4{\cdot}5H_2O$ at different concentrations (the final concentrations of 25, 50, and 100 µM). Then, these solutions were mixed with the equal volume of JEO and incubated at room temperature for 15 minutes.

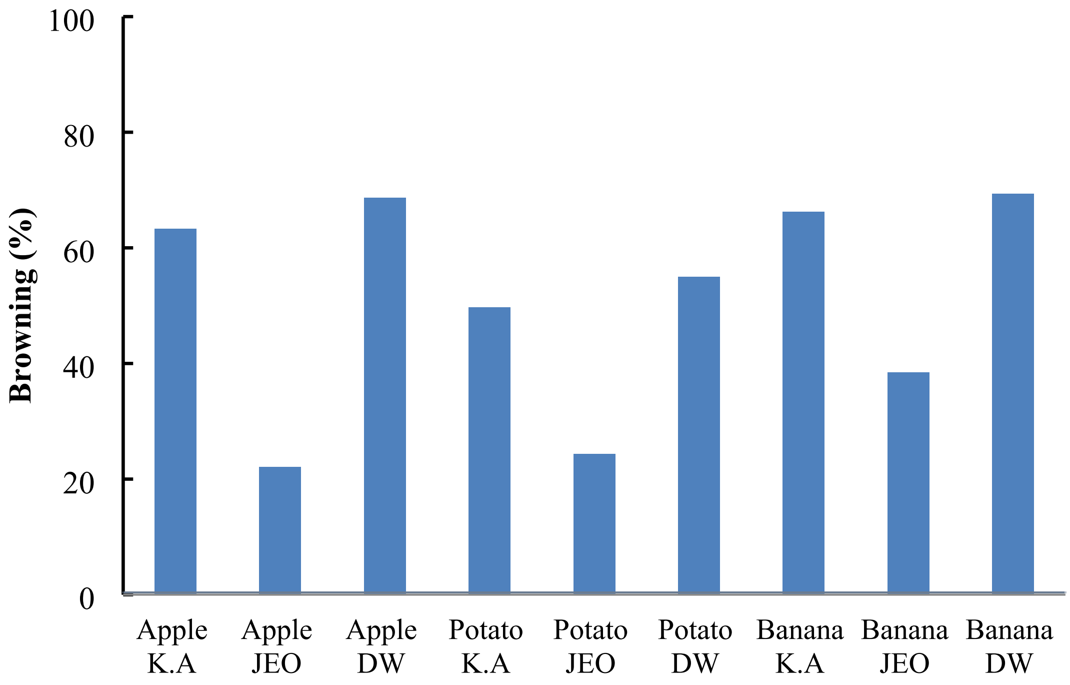

Fig. S3. Quantitative color analysis of the visual anti-browning results (**Fig. 3B**) of *Juniperus excelsa* essential oil (JEO) and kojic acid (K.A) on fresh-cut fruits (banana and apple) and vegetable (potato) using ImageJ software. The samples treated with distilled water (DW, as negative control) and kojic acid (K.A, as positive control), are shown for comparision.

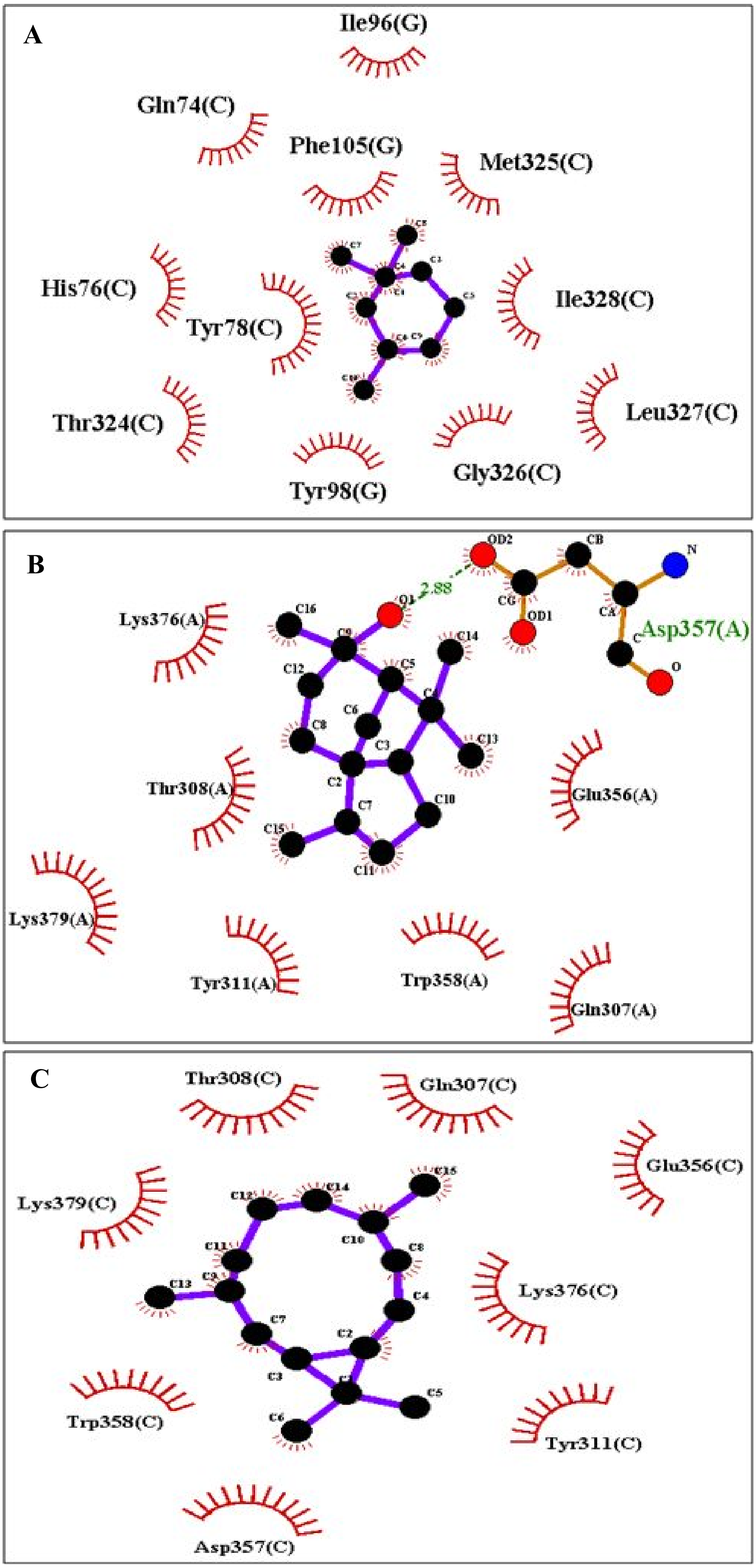
A
Ile96(G)
Gln74(C)
Phe105(G)
Met325(C)
His76(C)
Tyr78(C)
Ile328(C)
Thr324(C)
Leu327(C)
Tyr98(G)
Gly326(C)
B
Lys376(A)
2.88
Asp357(A)
Thr308(A)
Glu356(A)
Lys379(A)
Tyr311(A)
Trp358(A)
Gln307(A)
C
Thr308(C)
Gln307(C)
Glu356(C)
Lys379(C)
Lys376(C)
Trp358(C)
Tyr311(C)
Asp357(C)

Fig. S4. The results of molecular docking analyses of the predominant components of the essential oil (bicyclo [3.1.1] hept-2-ene, 3,6,6-trimethyl (A), cedrol (B), and (1S,2E,6E,10R)-3,7,11,11 tetramethylbicyclo [8.1.0] undeca-2,6-diene) (C) with mushroom tyrosinase. The images represent the results analyzed with Ligplot software for each ligand.

Table S1. The details of interactions between mushroom tyrosinase and bicyclo [3.1.1] hept-2-ene, 3,6,6-trimethyl. The bond distances are in angstrom (Å).

Number	Name	Category and Type	From chemistry	To chemistry	Distance (Å)
1	X:UNK0 C:ILE328	Hydrophobic Alkyl	X: UNK0	C:ILE328	5.38
2	X:UNK0:C8 C:MET325	Hydrophobic Alkyl	X:UNK0:C8	C:MET325	4.60
3	X:UNK0:C10 C:ILE328	Hydrophobic Alkyl	X:UNK0:C10	C:ILE328	4.68
4	C:TYR78 X:UNK0:C10	Hydrophobic Pi-Alkyl	C:TYR78	X:UNK0:C10	4.14
5	G:PHE105 X:UNK0	Hydrophobic Pi-Alkyl	G:PHE105	X:UNK0	7.57
6	G:PHE105 - X:UNK0:C10	Hydrophobic Pi- Alkyl	G:PHE105	X:UNK0:C10	6.39

Table S2. The details of interactions between mushroom tyrosinase and cedrol. The bond distances are in angstrom (Å).

Number	Name	Category and Type	From chemistry	To chemistry	Distance (Å)
1	A:ASP357:OD2 X:UNK0:O1	Unfavorable Acceptor-Acceptor	A:ASP357:OD2	X:UNK0:O1	5.12
2	X:UNK0:C13 A:TRP358	Hydrophobic Pi-Sigma	X:UNK0:C13	A:TRP358	4.41
3	A:LYS376 X:UNK0	Hydrophobic Alkyl	A:LYS376	X:UNK0	6.30
4	X:UNK0:C15 A:LYS376	Hydrophobic Alkyl	X:UNK0:C15	LYS376	4.64
5	X:UNK0:C15 A:LYS379	Hydrophobic Alkyl	X:UNK0:C15	A:LYS379	3.91
6	A:TRP358 X:UNK0:C13	Hydrophobic Pi- Alkyl	A:TRP358	X:UNK0:C13	5.11
7	A:TRP358 X:UNK0	Hydrophobic Pi-Alkyl	A:TRP358	X:UNK0	6.65

Table S3. The details of interactions between mushroom tyrosinase and 1S,2E,6E,10R)-3,7,11,11 tetramethylbicyclo [8.1.0] undeca-2,6-diene (Bicyclogermacrene). The bond distances are in angstrom (Å).

Number	Name	Category and Type	From chemistry	To chemistry	Distance (Å)
1	C:LYS376 X:UNK0	Hydrophobic Alkyl	C:LYS376	X:UNK0	5.20
2	X:UNK0 C:LYS376	Hydrophobic Alkyl	X:UNK0	C:LYS37 6	4.92
3	C:TRP358 X:UNK0:C13	Hydrophobic Pi-Alkyl	C:TRP358	X:UNK0: C13	5.23
4	C:TRP358 X:UNK0:C13	Hydrophobic Pi-Alkyl	C:TRP358	X:UNK0: C13	4.15

Table S4. The effect of JEO on the growth of gram-positive (*S. aureus*) and gram-negative (*E. coli*) strains was evaluated through the inhibition zones formation (diameters are in mm).

**Sample Volume (μl)**	**Inhibition zone (mm)**	
	Staphylococcus aureus	*Escherichia coli*
20	0	0
40	0	0
60	0	0
80	18	0
100	21	0
Positive control	28	20
Negative control	0	0

*Tetracycline (30 μg) and sterile water discs were used as the positive and negative controls, respectively.

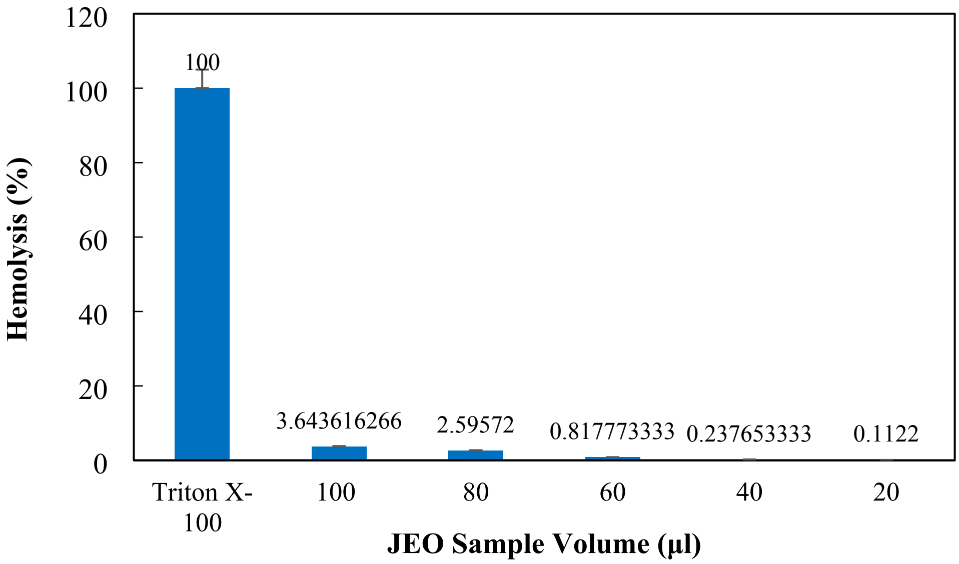

Fig. S5. The *in vitro* hemolytic effects of different concentrations of essential oil on human red blood cells were examined. The cells were treated with the essential oil for duration of 3 hours, and the data presented are the averages from a minimum of three independent experiments. Triton X-100 was utilized as the positive control in all assays and comparisons.
